# The Spatiotemporal Pattern of the Human Electroencephalogram at Sleep Onset After a Period of Prolonged Wakefulness

**DOI:** 10.3389/fnins.2019.00312

**Published:** 2019-04-03

**Authors:** Maurizio Gorgoni, Chiara Bartolacci, Aurora D’Atri, Serena Scarpelli, Cristina Marzano, Fabio Moroni, Michele Ferrara, Luigi De Gennaro

**Affiliations:** ^1^Department of Psychology, Sapienza University of Rome, Rome, Italy; ^2^Department of Biotechnological and Applied Clinical Sciences, University of L’Aquila, L’Aquila, Italy; ^3^IRCCS Santa Lucia Foundation, Rome, Italy

**Keywords:** sleep onset, sleep deprivation, EEG topography, oscillatory activity, BOSC

## Abstract

During the sleep onset (SO) process, the human electroencephalogram (EEG) is characterized by an orchestrated pattern of spatiotemporal changes. Sleep deprivation (SD) strongly affects both wake and sleep EEG, but a description of the topographical EEG power spectra and oscillatory activity during the wake-sleep transition after a period of prolonged wakefulness is still missing. The increased homeostatic sleep pressure should induce an earlier onset of sleep-related EEG oscillations. The aim of the present study was to assess the spatiotemporal EEG pattern at SO following SD. A dataset of a previous study was analyzed. We assessed the spatiotemporal EEG changes (19 cortical derivations) during the SO (5 min before vs. 5 min after the first epoch of Stage 2) of a recovery night after 40 h of SD in 39 healthy subjects, analyzing the EEG power spectra (fast Fourier transform) and the oscillatory activity [better oscillation (BOSC) detection method]. The spatiotemporal pattern of the EEG power spectra mostly confirmed the changes previously observed during the wake-sleep transition at baseline. The comparison between baseline and recovery showed a wide increase of the post- vs. pre-SO ratio during the recovery night in the frequency bins ≤10 Hz. We found a predominant alpha oscillatory rhythm in the pre-SO period, while after SO the theta oscillatory activity was prevalent. The oscillatory peaks showed a generalized increase in all frequency bands from delta to sigma with different predominance, while beta activity increased only in the fronto-central midline derivations. Overall, the analysis of the EEG power replicated the topographical pattern observed during a baseline night of sleep but with a stronger intensity of the SO-induced changes in the frequencies ≤10 Hz, and the detection of the rhythmic activity showed the rise of several oscillations at SO after SD that was not observed during the wake-sleep transition at baseline (e.g., alpha and frontal theta in correspondence of their frequency peaks). Beyond confirming the local nature of the EEG pattern at SO, our results show that SD has an impact on the spatiotemporal modulation of cortical activity during the falling-asleep process, inducing the earlier emergence of sleep-related EEG oscillations.

## Introduction

Sleep is widely considered as a local and use-dependent process, since regional patterns of activation and deactivation coexist in the brain during sleep, and recent evidence suggests that large regional heterogeneities also occur during specific transitional states (for review see Ferrara and De Gennaro, 2011; Siclari and Tononi, 2017). Indeed, the sleep onset (SO) period represents a complex phenomenon of transition between two different functional states (i.e., wakefulness and sleep) of the brain in which the fluctuation of consciousness seems to result from topographically heterogeneous cortical activities (Gorgoni et al., 2019). In particular, the falling-asleep process is characterized by: (a) wide regional and temporal frequency-specific changes in the scalp electroencephalogram (EEG) (De Gennaro et al., 2001a,b; Marzano et al., 2013; Siclari et al., 2014); (b) asynchronies between cortical and deep structures in the appearance of sleep rhythms (Magnin et al., 2010; Sarasso et al., 2014); (c) modifications of the dynamic interactions between functionally specialized regions (De Gennaro et al., 2004; Larson-Prior et al., 2009; Vecchio et al., 2017; Fernandez Guerrero and Achermann, 2018); (d) changes in cerebral metabolic activity (Kjaer et al., 2002; Kotajima et al., 2005; Horovitz et al., 2008).

At the scalp level, the human EEG power spectra at SO show the cohabitation of wake-like and sleep-like activities, with an earlier synchronization of the fronto-central areas expressed by the anterior increase of the slow-wave activity (SWA), a temporo-occipital diffusion in the theta frequency range, a shift from a posterior to an anterior prevalence of the alpha activity, an increase in the sigma frequency range mostly at a centro-parietal level and a generalized decrease in the beta frequency (Marzano et al., 2013). The direct assessment of the genuine oscillatory nature of the EEG at SO by means of the application of the Better OSCillation detection method (BOSC analysis; Caplan et al., 2001; Whitten et al., 2011) suggested that the pattern of synchronization during SO apparently does not include alpha and frontal theta oscillations (Marzano et al., 2013). However, we cannot exclude that these unexpected findings were due to the short window considered for the SO period (i.e., the two 5-min intervals before and after the first epoch of stage 2), which could make not viable the observation of specific EEG oscillatory activities with a more delayed build-up.

What is still missing is the description of the spatial and temporal modification of the EEG power spectra and rhythmic oscillations at SO after a period of prolonged wakefulness. It is well-known that sleep deprivation (SD) strongly affects the human EEG during wake (Finelli et al., 2000; Tinguely et al., 2006; De Gennaro et al., 2007; Gorgoni et al., 2014) and recovery sleep (Cajochen et al., 1999; Finelli et al., 2001; Tinguely et al., 2006; Marzano et al., 2010), mainly with a generalized increase of the slowest frequencies. Moreover, SD also has an influence on the EEG activity during the transition from recovery sleep to wake (i.e., the process of awakening) (Tassi et al., 2006; Gorgoni et al., 2015). Surprisingly, albeit several findings underlined that a period of prolonged wakefulness induced marked changes in functional coupling (De Gennaro et al., 2005), effective connectivity (Fernandez Guerrero and Achermann, 2018) and in the brain dynamics assessed with EEG source localization (Fernandez Guerrero and Achermann, 2019) during the wake-sleep transition, the effect of SD on the SO-related scalp topographical changes in the EEG power and oscillatory activity has been not systematically studied. Since SD induces (a) an acceleration of the baseline electrophysiological dynamics at SO (De Gennaro et al., 2005; Fernandez Guerrero and Achermann, 2019) and (b) an increase of the delta, theta and alpha activity during REC sleep (Marzano et al., 2010), we hypothesize that the higher homeostatic sleep pressure should determine an earlier onset of oscillatory EEG changes during the falling-asleep process that usually occurs later during a night of sleep under normal conditions. If this is true, we should observe a greater concordance between spectral and oscillatory data: while in the BSL night the spectral power and the oscillatory activity differed because no post-SO changes were observed in the alpha and frontal theta oscillatory peaks (Marzano et al., 2013), SD should provoke an earlier build-up of the alpha and theta oscillations, mirroring the increase in the spectral power in these frequency bands.

The aim of the present study was to assess the wake-sleep transition after 40 h of SD in the same 40 healthy subjects previously investigated at BSL (Marzano et al., 2013). We assessed the single-Hz changes in spatial EEG at SO and the temporal dynamics of the frequency bands before and after SO. Subsequently, we used the BOSC method which measures oscillatory activity within an EEG signal containing a non-rhythmic portion, in order to detect sleep EEG oscillations before and after SO, and then their spatial and temporal variations have been investigated.

## Materials and Methods

### Participants

Data for the present analyses were obtained by the sample of previous studies (Marzano et al., 2010, 2013). Forty right-handed healthy subjects (20 males and 20 females; age range = 18–29, mean age = 23.8 ± 2.88 years) were selected from a university student population. The inclusion criteria were: normal sleep duration and schedule (habitual sleep time: midnight-8:00 am ± 1 h), no daytime nap habits, no excessive daytime sleepiness, no other sleep, medical, neurological or psychiatric disorder, as assessed by a 1-week sleep log and by a clinical interview. Participants were required to avoid napping throughout the experiment; compliance was controlled by actigraphic recordings (AMI Mini motion logger). One subject was excluded from the analyses because he did not show artifact-free epoch before the SO. Therefore, analyses were conducted on 39 subjects.

All subjects gave their written informed consent. The study was approved by the Institutional Ethics Committee of the Department of Psychology of “Sapienza” University of Rome and was conducted in accordance with the Declaration of Helsinki.

### Procedure

After 2 weeks of regular sleep/wake habits monitored with sleep log and (in the last 2 days before the beginning of the study) actigraphic recordings, subjects participated in a sleep/wake protocol across four consecutive days and nights. Sleep was recorded during the first night (adaptation), the second night [baseline (BSL)], and the fourth night [recovery (REC)]. After awakening from BSL sleep, a protocol of 40-h SD started at 10.00 am. For the purposes of the present study, the main analyses have been performed on the REC night, while the BSL condition has been considered only for the comparison of polysomnographic (PSG) measures and post- vs. pre-SO changes in the EEG power topography.

Sleep recordings have been carried out in a sound-proof, temperature-controlled room. The subjects’ sleep was undisturbed, started at midnight, and ended after 7.5 h of accumulated sleep (as visually checked online by expert sleep researchers).

### Polysomnographic Recordings

An Esaote Biomedica VEGA 24 polygraph was used for PSG recordings. EEG signals were analogically filtered (high-pass filter at 0.50 Hz and antialiasing low-pass filter at 30 Hz [-30 dB/octave]). The 19 unipolar EEG derivations of the international 10–20 system (Fp1, Fp2, F7, F8, F3, F4, Fz, C3, C4, Cz, P3, P4, Pz, T3, T4, T5, T6, O1, O2) were recorded from scalp electrodes with averaged mastoid reference. The submental electromyogram (EMG) was recorded with a time constant of 0.03 s. Bipolar horizontal electrooculogram (EOG) was recorded from electrodes placed approximately 1 cm from the medial and lateral canthi of the dominant eye with a time constant of 1 s. The impedance of these electrodes was kept below 5 kOhm.

### Data Analysis

#### PSG and Quantitative Analyses of EEG Signals

The midline central EEG derivation (Cz), EMG, and EOG were used to visually score sleep stages in 12-s epochs, according to the standard criteria (Rechtschaffen and Kales, 1968). PSG measures were submitted to paired *t*-tests comparing BSL and REC nights (alpha level = 0.05).

The polygraphic signals (19 EEG channels, EOG, and EMG) were analog-to-digital converted online with a sampling rate of 128 Hz and stored on the disk of a personal computer. We considered the 0.50- to 25.00-Hz frequency range, computing power spectra by a fast Fourier transform (FFT) routine for 4-s periodograms. Spectra from three consecutive 4-s epochs were averaged to allow alignment with the visual scoring of sleep stages, based on 12-s epochs. EEG topography was evaluated by comparing the 5-min pre-SO vs. post-SO intervals. Ocular and muscle artifacts were carefully excluded offline by visual inspection. Epochs in which eyes were open were also excluded. After the exclusion of the artifacts, the mean duration of the REC falling-asleep period (pre- and post-SO) included in the spectral analysis was 5.95 ± 0.98 min (range: 3.4–8.8 min). Individual time series of EEG power values were aligned as a function of the first epoch of sleep defined on the basis of the appearance of the first K-complex or sleep spindle. Data analysis was mostly performed with the software package MATLAB (The Mathworks, Inc., Natick, MA, United States) and its signal analysis and statistics toolbox.

#### Single-Hz EEG Topography

Data were reduced to a 1-Hz bin width by collapsing four adjacent 0.25-Hz bins before statistical analyses. The only exception was the 0.50- to 1.00-Hz bin, for which two adjacent 0.25-Hz bins were collapsed. The bins were referred to and plotted by the center frequency included in our study (e.g., the 2-Hz bin referred to the averaged values of the following bins: 2.00, 2.25, 2.50, and 2.75 Hz). The log-transformed EEG power values for each 1-Hz frequency bin of the REC night were considered as dependent measures and compared in the 5-min pre- and post-SO intervals by paired *t*-tests. As a standard procedure for EEG power, this log-transformation reduces violations of normality.

With the aim to assess the influence of SD on the magnitude of the SO-related topographical changes, we also compared the post- vs. pre-SO ratio of the raw EEG power between the REC and the BSL night for each frequency bin. Such comparison was computed using the Wilcoxon signed-rank tests, since only 52% of our data had a normal distribution (Lilliefors test). The difference between REC and BSL post- vs. pre-SO ratio has been calculated with the aim to depict the direction of the findings.

For every statistical comparison performed on topographical data, the Bonferroni correction for multiple comparisons was applied.

#### Time Course of the EEG Frequency Bands

Due to the variable length of the pre-SO and post-SO intervals and of the first sleep cycle, we adopted for the REC night the procedure previously used for the analysis of the time course during SO at BSL (Marzano et al., 2013) to make the individual time courses comparable: (1) the individual time courses were aligned as a function of the first spindle or K-complex; (2) the time series of 12-s epochs during the pre-SO interval were divided into five segments, while the post-SO time series were divided into 20 segments (percentiles); and (3) we removed epochs with muscle, movement, or ocular artifacts and averaged individual time courses across subjects. In this way, each pre- and post-SO interval represented a fifth and a 20th percentile of the total pre- and post-sleep intervals, respectively. Neither skipped first REM sleep episodes nor SO REM sleep episodes were present in our recordings.

For the five time intervals preceding and following SO, EEG power maps were computed for the following frequency EEG bands: delta (0.50–4.75 Hz), theta (5.00–7.75 Hz), alpha (8.00–11.75 Hz), sigma (12.00–15.75 Hz), and beta (16.00–24.75 Hz). Power maps at the 10th, 15th, and 20th time intervals were also calculated, with the aim to provide a synoptic description of the kinetics of EEG topography across the first sleep cycle.

#### Detection of Oscillatory Activity

As previously observed (Marzano et al., 2013), the FFT algorithm of the EEG signal does not necessarily imply an underlying oscillatory activity at that specific frequency. The FFT is mainly designed for stationary and regular signals and is characterized by a limited time-frequency resolution. However, the EEG pattern during the SO period is more likely characterized by changes in oscillatory (non-stationary) activity. For this reason, we also applied to the EEG signals of the REC night the BOSC detection method introduced by Caplan et al. (2001). BOSC is considered a powerful method to detect oscillatory neural activity minimizing bias across frequencies, electrodes, tasks, electrophysiological states and species (Whitten et al., 2011). This method is aimed to detect oscillatory activity within an EEG signal containing a non-rhythmic portion, considering the functional form of the background non-rhythmic portion of the signal and revealing segments of the recording that significantly deviate from the spectral characteristics of the background. We recently applied the BOSC method to detect oscillatory activity during wake (D’Atri et al., 2015), NREM and REM sleep (Marzano et al., 2011; Moroni et al., 2012; Scarpelli et al., 2015) and in the wake-sleep transition at BSL (Marzano et al., 2013).

The analysis was separately performed for each frequency of interest (in the 0.50–24.25-Hz range), cortical derivation and 5-min time segment before and after SO, and then averaged across subjects. For each frequency, an oscillatory episode was defined as an epoch longer than a duration threshold (DT, set to three cycles in our analysis) during which wavelet power at frequency exceeded a power threshold (PT). BOSC identifies a power increase, above PT, of a minimum duration (DT), rejecting transient events that are not oscillatory but can induce increases in the spectral power that may be erroneously considered as rhythmic activity. This PT threshold was chosen as follows in the selected segments before and after SO: (1) the EEG was wavelet transformed (Morlet wavelet, window = 6 cycles) at 47 logarithmically spaced frequencies in the 0.50- to the 24.25-Hz range. The average of the log-transform of these wavelet values yielded the wavelet power spectrum; (2) the background noise spectrum assumed the form Power(*f*) = A*f*^-α^. We used two different fits of background window for pre- and post-SO periods. The estimate of this background has been obtained by fitting the observed spectrum at each electrode with a linear regression in the log to log units. The background at *f*^∗^ has been estimated on the mean of its corresponding χ^2^(2) probability distribution function. The power threshold (*P*_T_) was set to the 95th percentile of the theoretical probability distribution. The proportion of time in which significant oscillations were detected before and after SO is termed P_episode_ (Caplan and Glaholt, 2007).

As previously done for the BSL night (Marzano et al., 2013), to provide a statistical assessment of the SO-related topographical changes in the oscillatory activity during the REC night, for the frequency peak of each band we compared the proportion of time in which significant oscillations were detected in the pre-SO and post-SO periods, separately for each cortical derivation. Since the majority of the derivations (74%) showed a normal distribution (Lilliefors test) and given the robustness of parametric *t*-tests to violation of normality (Siclari et al., 2017), we used paired *t*-tests.

Finally, the time course of the values of P_episode_ resulting from five pre-SO and post-SO time intervals was reported for each frequency and scalp location.

## Results

### PSG Measures

Table 1 reports the results of the comparisons (paired *t*-tests) between PSG measures of BSL and REC nights. The macrostructural variables of sleep point to a pattern of significant differences between BSL and REC nights, representing the typical consequences of a night of SD: REC night was characterized by a shortening in the latency of NREM sleep stages, by an increase of the percentage of SWS and a decrease of stage 1, by a decreased percentage of WASO, number of awakenings and arousals; sleep efficiency was increased, while TBT was significantly decreased; with respect to REM sleep, its latency did not change, while its amount during REC sleep decreased.

**Table 1:** Polysomnographic measures.

Variables	*BSL*		*REC*		*t*(1,38)	*p*
	Mean	*SD*	Mean	*SD*		
Stage 1 latency (min)	6.73	5.81	1.93	2.09	5.8	**0**.**0001**
Stage 2 latency (min)	11.40	11.46	3.27	2.37	4.8	**0**.**0001**
Stage 1 (%)	6.36	2.94	2.85	1.71	8.84	**0**.**0001**
Stage 2 (%)	59.25	6.76	58.65	8.48	0.6	0.55
Stage 3 (%)	7.75	3.77	11.87	3.89	7.73	**0**.**0001**
Stage 4 (%)	2.21	3.27	5.89	6.01	6.32	**0**.**0001**
SWS (%)	9.96	6.06	17.76	7.71	11.94	**0**.**0001**
REM (%)	24.42	4.42	20.74	5.73	4.15	**0**.**0002**
WASO (min)	26.44	19.30	11.69	7.66	4.96	**0**.**0001**
Awakenings (#)	28.67	10.74	20.51	7.61	5.61	**0**.**0001**
Arousals (#)	35.54	17.79	26.18	18.74	3.16	**0**.**003**
TST (min)	440.90	39.03	449.30	20.27	1.49	0.14
TBT (min)	476.98	40.69	463.61	21.98	2.21	**0**.**03**
SEI % (TST/TBT)	92.51	4.21	96.92	1.72	6.51	**0**.**0001**

*Means and standard deviations (SD) of the PSG variables, during baseline (BSL) and recovery (REC) nights in 39 subjects. The results of paired *t*-tests are also reported. SWS, slow-wave sleep; REM, rapid eye movement; WASO, wake after sleep onset; TST, total sleep time; TBT, total bed time; SEI, sleep efficiency index. Significant results (*p* < 0.05) were reported in bold*.

### Single-Hz EEG Topography

**Figure 1:**
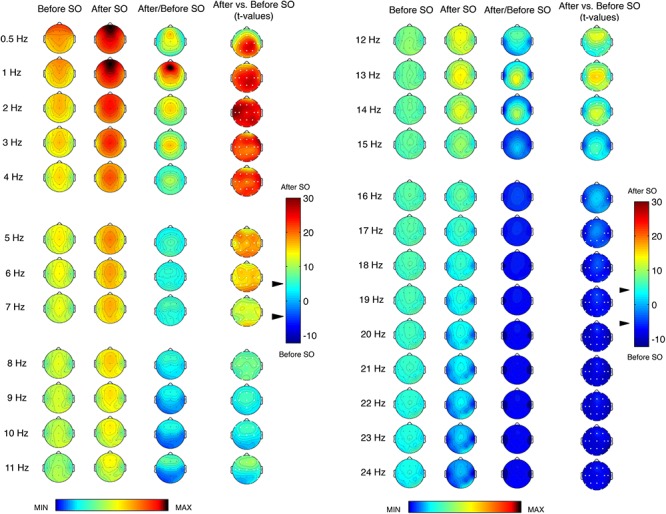
Single-Hz electroencephalographic (EEG) topography (left side, frequency range = 0.50–11 Hz; right side, frequency range = 12–24 Hz) at sleep onset (SO) after a period of prolonged wakefulness. In both halves of the figure, the first two columns show the topographic distribution of absolute EEG power in the 5-min intervals before SO and after SO, respectively. The maps were scaled between minimal (min) and maximal (max) values calculated for all frequencies and derivations in before SO and after SO periods. The third column shows the relative EEG changes expressed as the ratio between after SO and before SO periods. The maps were scaled between min and max values in before SO and after SO periods. The fourth column depicts the topographical statistical EEG power differences (assessed by paired *t*-tests) between these 5-min periods. Values are expressed in *t*-values: positive *t*-values indicate a prevalence of the after SO period and vice versa. The two-tailed level of significance is indicated by the arrow in correspondence of the *t*-values color bar (*p* ≤ 0.0005 corresponding to *t* ≥ 3.79 after the Bonferroni correction). White dots indicate significant differences after the Bonferroni corrections. Values are color-coded and plotted at the corresponding position on the planar projection of the scalp surface and are interpolated (biharmonic spline) between electrodes. The maps are based on the 19 unipolar EEG derivations of the international 10–20 system with averaged mastoid reference, and they are plotted for each frequency Hz bin in the 0.50- to the 24.75-Hz range.

Figure 1 shows the topographic distribution of the EEG power before and after SO during the REC night and their comparison (ratio and *t*-values). We found a significant (*p* ≤ 0.0005 corresponding to *t* ≥ 3.79 after the Bonferroni correction) differences between pre- and post-SO in all cortical derivations in the ≤8 Hz frequency range, in the direction of a power increase after SO. The EEG power under 2 Hz exhibited a clear prevalence in the anterior regions both before and after SO, and the widespread significant increase of EEG power after SO in this frequency range reached its maximum in the left fronto-temporal area (2 Hz bin) and the centro-parietal derivations (0.5 and 1 Hz bins). The EEG topography in the 3–4 Hz bins showed a central prevalence before and after SO, but the statistical comparisons pointed to frontal maxima. The theta frequency range (5–7 Hz) also exhibited a maximal EEG power in the central area both before and after SO, and the *t*-values maps pointed to a larger post-SO increase in the occipital region.

The EEG power topography in the 8–12 Hz frequency range was characterized by a transition from a posterior prevalence before SO to a frontal prevalence after SO. The statistical comparisons showed a post-SO significant increase in all cortical areas at 8 and 12 Hz, and in the fronto-central areas in the frequency bins between 9 and 11 Hz, extended to the temporal (9 Hz: T3; 11 Hz: T3, T4, T6) and parietal (9 Hz: Pz) derivations. All these frequency bins showed a maximum post-SO increase in the frontal region.

The sigma frequency range (13–15 Hz) was characterized by marked differences between pre- and post-SO, due to the scoring of SO as the first epoch of stage 2. Such differences, all in the direction of a post-SO increase, reached the statistical significance in all cortical derivations at 13 Hz, in a wide number of derivations at 14 Hz (C3, C4, Cz, F3, F4, Fz, O1, O2, P3, P4, Pz, T5, T6) and in some centro-parietal locations at 15 Hz (C3, Cz, P3, Pz). Prevalence in the midsagittal centro-parietal areas was detectable in all these frequency bins.

While the significant difference between pre- and post-SO in the ≤15 Hz frequencies pointed to a power increase after SO, in the ≥16 Hz frequency range we observed a change in the direction of these differences, that is a post-SO power decrease. In the 16 Hz bin, such power decrease reached the statistical significance only in two temporal derivations (T3, T4). The 17 and 18 Hz frequencies showed a widespread significant power decrease after SO, and in the ≥19 Hz frequencies these significant changes concerned to all cortical derivations, without a specific topographical prevalence.

**Figure 2:**
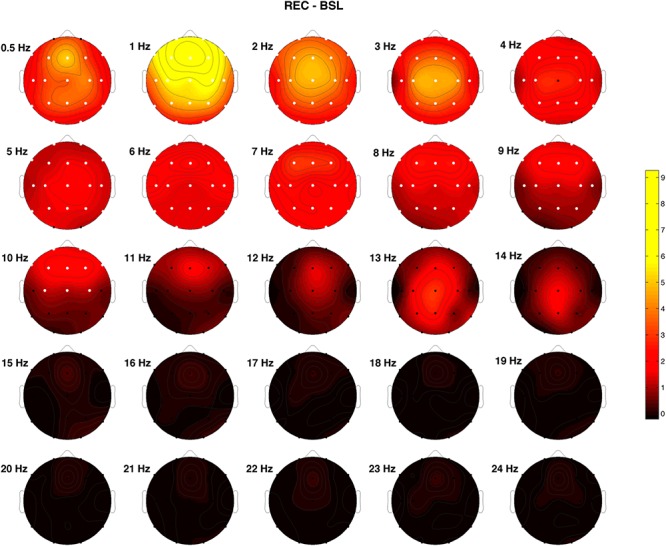
Topographical EEG power differences between recovery (REC) and baseline (BSL) conditions performed on the relative EEG changes expressed as the ratio between after SO and before SO periods (post-SO/pre-SO). Positive values indicate a higher post-SO/pre-SO ratio in the REC condition and vice versa. White dots indicate significant differences at the Wilcoxon signed-rank tests after the Bonferroni corrections (*p* ≤ 0.0003). Values are color-coded and plotted at the corresponding position on the planar projection of the scalp surface and are interpolated (biharmonic spline) between electrodes. The maps are based on the 19 unipolar EEG derivations of the international 10–20 system with averaged mastoid reference, and they are plotted for each frequency Hz bin in the 0.50- to 24.75-Hz range.

Figure 2 shows the difference between REC and BSL night concerning the post-SO/pre-SO ratio of the raw EEG power. White dots indicate the significant differences at the Wilcoxon signed-rank tests after Bonferroni correction (*p* ≤ 0.0003). A significantly higher ratio in the REC condition was observed in the frequency bins ≤9 Hz in all (1–3, 6 and 7 Hz) or almost all (0.5, 4 and 5, 8 and 9 Hz) cortical derivations, and in the 10 Hz bin at a fronto-central level (F1, F2, F3, F4, F7, F8, Fz, C3, C4, Cz), representing an index of higher synchronized activity (i.e., higher sleep pressure) during the REC night.

### Time Course of EEG Frequency Bands

**Figure 3:**
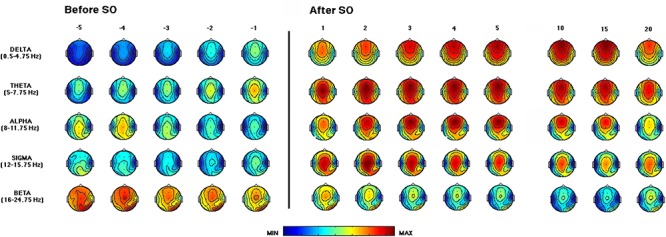
Time course of EEG frequency bands during the recovery (REC) night. From the left, topographic EEG shows changes across the interval preceding SO (i.e., from the lights-off to the first epoch of sleep). The power maps obtained by subdividing the pre-SO interval into five equal parts (from the -fifth to -first). Data were calculated for each subject and then averaged across subjects. After the vertical line, indicating the first epoch of stage 2 sleep, the first five intervals after SO (from the first-fifth) are plotted. The last three columns on the right side show power maps at the 10th, 15th, and 20th time intervals calculated across the first sleep cycle. Values are color-coded and plotted at the corresponding position on the planar projection of the scalp surface and are interpolated (biharmonic spline) between electrodes. The maps are based on the 19 unipolar EEG derivations of the international 10–20 system with averaged mastoid reference. Maps are plotted for the following EEG bands: delta (0.50–4.75 Hz), theta (5.00–7.75 Hz), alpha (8.00–11.75 Hz), sigma (12.00–15.75 Hz), and beta (16.00–24.75 Hz).

Figure 3 depicts the time course of the EEG power across the SO point and during the first NREM sleep episode of the REC night. The delta activity showed a gradually increasing fronto-central prevalence in the pre-SO intervals. After SO, a phenomenon of anteriorization of this frequency range was clearly visible, with maximal power values over the midsagittal frontal derivation, that decreased only in close proximity to the end of the first NREM episode (i.e., the time interval just before the 20th percentile).

The theta frequency range was characterized by a growing fronto-central prevalence before and after SO. An increase in the occipital area was visible in close proximity to SO and persisted across the first NREM sleep episode.

The alpha activity showed a centro-posterior prevalence in the pre-sleep intervals that progressively decreases, while after SO we observed a gradual increase in this frequency range, particularly in fronto-central areas, that showed a reduction only at the end of the first NREM episode.

The sigma frequency range exhibited a marked increase after SO, with a clear centro-parietal localization, as expected since the operational definition of SO.

The beta activity showed fronto-central and occipital prevalence, and its power progressively decreased across the SO point.

### Detection and Topography of Oscillatory Activity

**Figure 4:**
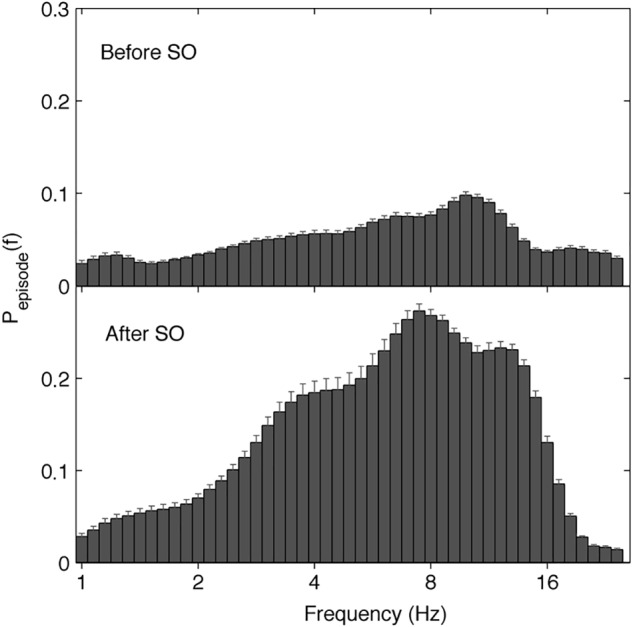
Electroencephalographic oscillations averaged across the whole derivations at SO during the recovery (REC) night. The figure plots the mean proportion of time (P_episode_ [f]) of the EEG before (upper panel) and after (lower panel) SO in which oscillations were detected at each frequency. The detection of oscillations has been made by the better oscillation detection method (see section Materials and Methods) on the 19 EEG electrodes. Error bars denote interlocation variability. Units of frequency are expressed in Hz and are plotted in 47 logarithmically spaced frequency values in the 0.50- to 24.25 Hz frequency range.

**Figure 5:**
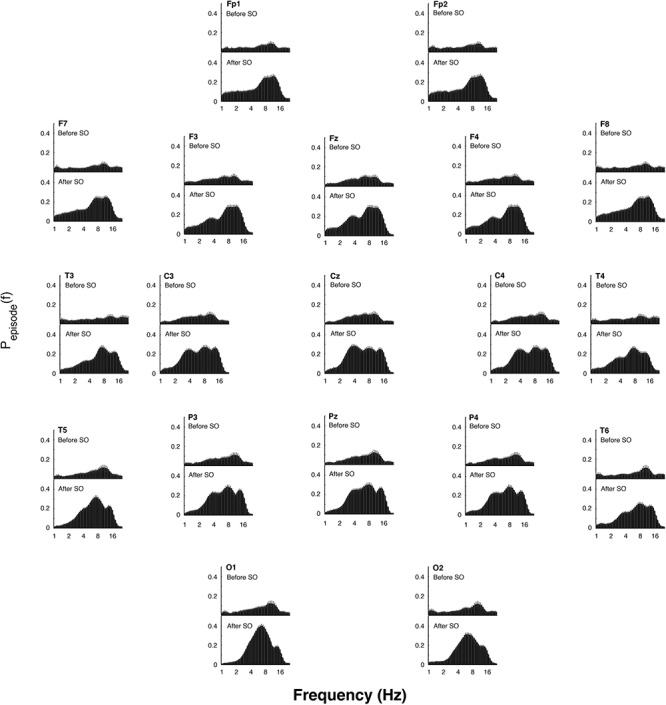
Topographic distribution of the EEG oscillations detailed by the better oscillation method during the 5-min segments before and after SO (upper and lower panels, respectively) in the recovery (REC) night. The mean proportion of time (P_episode_ [f]) of the EEG before and after SO in which oscillations were detected for each scalp location is plotted. Error bars denote intersubject variability. Units of frequency are expressed in Hz and are plotted in 47 logarithmically spaced frequency values in the 0.50- to 24.25 Hz frequency range.

The EEG oscillatory activity averaged across all the derivations during the SO of the REC night is illustrated in Figure 4, while Figure 5 depicts its topographical modulation. Pre-sleep EEG was characterized by a prevalent alpha oscillatory activity, with a peak at 9.85 Hz, followed by the theta and beta activity, peaking at 6.06 and 18.38 Hz, respectively. The oscillation of the delta activity was already present. In the post-SO period, the prevalence of the theta range was visible, with a peak at 7.46 Hz, followed by sigma (peak at 12.12 Hz) and delta (peak at 3.73 Hz) activity. At a topographical level (Figure 5), pre-SO alpha showed a prevalence in posterior areas, and post-SO theta activity had a clear occipital predominance. Delta oscillatory activity ≤ 2 Hz had a frontal predominance after SO, while at 3–4 Hz it was more evident at central areas in both pre- and post-SO conditions.

**Figure 6:**
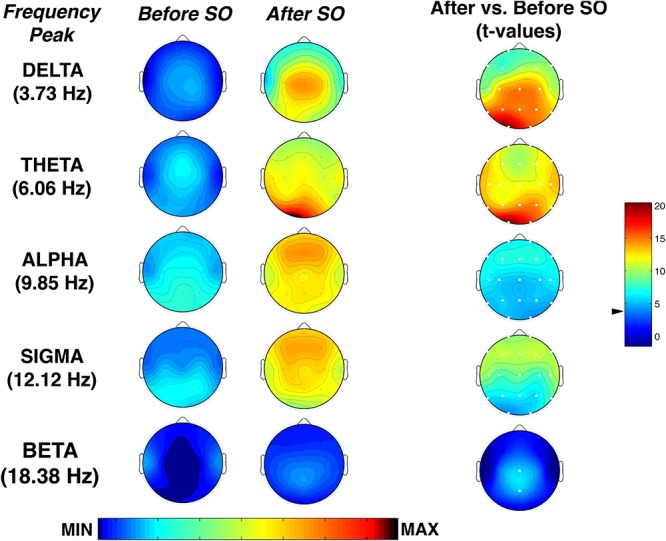
Topographic distribution of the frequency peak of oscillatory activity within each EEG frequency bands at SO during the recovery (REC) night. From the left, the first two columns show the topographic distribution of the mean proportion of time in which oscillations were detected (P_episode_ [f]) in correspondence of five selected frequencies of interest (3.73, 6.06, 9.85, 12.02, 18.38 Hz) among the five frequency bands (delta, theta, alpha, sigma, beta) in the 5-min intervals before SO and after SO, respectively. The maps were scaled between minimal (min) and maximal (max) values in the before SO and after SO periods. The first column on the right side shows topographical statistical P_episode_ differences (assessed by paired *t*-tests) between after SO and before SO periods. Values are expressed in *t*-values: positive *t*-values indicate a prevalence of the after SO over the before SO period, and vice versa. The two-tailed level of significance is indicated by the arrow in correspondence of the *t*-values color bar (*p* ≤ 0.001 corresponding to *t* ≥ 3.55 after Bonferroni correction). White dots indicate significant differences after the Bonferroni corrections. Values are color-coded and plotted at the corresponding position on the planar projection of the scalp surface and are interpolated (biharmonic spline) between electrodes. The maps are based on the 19 unipolar EEG derivations of the international 10–20 system with averaged mastoid reference.

Figure 6 shows the topographical maps of the comparison (*t*-test) between pre-SO and post-SO intervals in the frequency peak of each band, concerning the proportion of time in which significant oscillations were detected. Results showed a significant (*p* ≤ 0.001 corresponding to *t* ≥ 3.55 after Bonferroni correction) increase of all frequency peaks after SO in the whole scalp topography, except the beta band that significantly increased only in the midline centro-parietal derivations (Cz; Pz). The delta and theta activity showed a prevalence in posterior areas, reaching their maximum in the left occipital derivation, while alpha and sigma activity showed a more evident increase in the frontal locations.

### Time Course of the Oscillatory Activity

**Figure 7:**
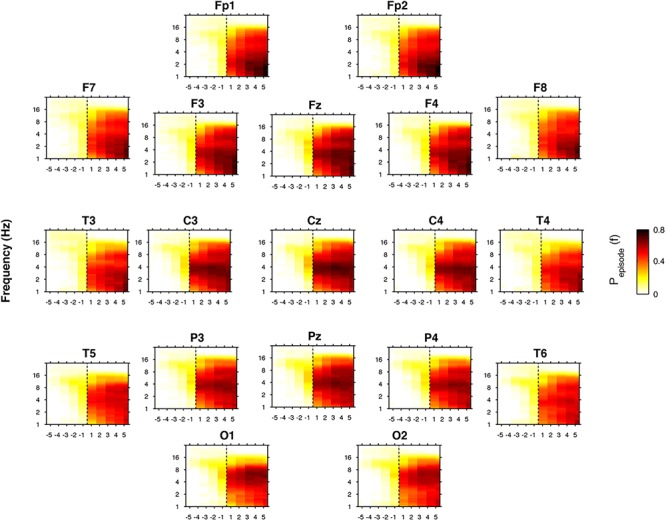
Time course of oscillatory activity at SO during the recovery (REC) night. Topographic time-frequency plots of the five intervals before (from the fifth-first) and after (from the first-fifth) SO, averaged across subjects at the 19 scalp derivations. The pre- and post-SO intervals were divided into five equal parts as in Figure 2. Data were calculated for each subject and then were averaged across subjects. Darker colors (red brown) are indicative of increase oscillatory (P_episode_) activity for a specific frequency of oscillation in the 0.50- to 24.25 Hz frequency range. SO is indicated by black dotted line.

Figure 7 depicts the time course of the oscillatory activity across the SO point of the REC night. The pre-sleep period was characterized by a generalized slight prevalence of the alpha oscillations, that gradually shift in the direction of a theta predominance just before SO, particularly evident in the cortical derivations along the midline. After SO, a wide increase of the oscillatory activity in the frequency bands between delta and sigma was observed, albeit with different topography and intensity. The occipital regions were characterized by an increasing post-SO prevalence of the theta oscillations, progressively extended to the delta range. In the parietal area, the sleep period was steadily dominated by delta and theta oscillations, with a progressive increase in alpha and sigma activities and in the slowest frequency bins. According to an anteroposterior gradient, delta oscillations dominated the post-SO period in frontal and central areas and became stronger with time. Alpha and sigma activities showed a wide and progressive increase during the sleep period, while beta oscillations mostly disappeared, albeit the slowest bins in the beta rage exhibited a stable level after SO.

## Discussion

To the best of our knowledge, this is the first study to describe the complete topography of the EEG power spectra and oscillatory activity across the wake-sleep transition after SD.

The spatio-temporal dynamic of the EEG power at SO after prolonged wakefulness substantially replicated findings in the wake-sleep transition during the BSL night in the same sample (Marzano et al., 2013), suggesting that SD does not alter the topographical pattern of the EEG power at SO. Interestingly, also the assessment of effective connectivity during the wake-sleep transition shows changes after SO qualitatively similar in the BSL and REC conditions (Fernandez Guerrero and Achermann, 2018). On the other hand, the direct comparison between REC and BSL revealed a generalized increase after SD of the post- vs. pre-SO ratio of the EEG power in the bins ≤9 Hz, that became fronto-centrally localized at 10 Hz. This finding suggests that a period of prolonged wakefulness increases the intensity of the SO-induced topographical EEG changes with an exacerbation of the EEG synchronization process, representing a sign of the higher homeostatic sleep need, in the absence of strong differences in the higher frequencies.

Concerning the analysis of the rhythmic oscillations, we found that SD had an influence on the modulation of the oscillatory activity during the falling-asleep process. Considering the average oscillatory activity across derivations, we found in the REC night that the theta rhythm was prevalent after SO, followed by sigma and delta rhythms, while the post-SO period at BSL (Marzano et al., 2013) was characterized by a dominant sigma oscillatory activity. These phenomena were topographically and temporally modulated, substantially confirming the EEG power findings. The topographic distribution of the frequency peaks of oscillatory activity showed a generalized increase after SO in all frequency bands from delta to sigma, albeit with different predominance (centro-posterior for delta activity, occipital for theta activity, anterior for alpha and sigma rhythms), while beta rhythm significantly increased only in the midline centro-parietal derivations. As expected, the procedure of SD induced an increase of alpha and frontal theta oscillatory activity in their frequency peaks after SO, not observed in normal conditions (Marzano et al., 2013), showing more consistent results between FFT and BOSC analysis compared to BSL. Our results support the hypothesis that the short window considered for the SO period (i.e., the two 5-min intervals before and after the first epoch of stage 2) may make difficult to detect several EEG oscillatory activities with a more delayed build-up (alpha and frontal theta) during a normal night of sleep, while the increased homeostatic sleep pressure after prolonged wakefulness leads to an earlier onset of such sleep-related rhythmic oscillations.

### Delta Activity

Delta power exhibited its classical fronto-central prevalence and a strong and generalized increase after SO, with the time course of this effect pointing to a progressive anteriorization that begins before the SO point. Changes in this frequency band represent the most prominent and fastest SO-related EEG power modifications, confirming and extending to a REC condition the previous findings in a normal night of sleep (De Gennaro et al., 2001a,b; Marzano et al., 2013).

The comparison between BSL and REC conditions showed a global increase of the post- vs. pre-SO ratio in the delta frequency bins, consistent with the consolidated role of sleep SWA as the marker of homeostatic sleep need after prolonged wakefulness (for a review see Achermann and Borbély, 2017).

The assessment of the oscillatory activity substantially confirmed the results obtained with the FFT, revealing a generalized enhancement of delta rhythm in its frequency peak during sleep, albeit with a peculiar maximum increase in the occipital area. However, the detailed topographical distribution of the EEG oscillations (Figure 5) showed that frontal, central and parietal areas exhibited a genuine peak of the delta oscillations after SO, while in the occipital region the great oscillatory activity around 3.73 Hz appeared as a by-product of the prevalent theta peak.

It is worth noting that, according to several findings, the SO process seems characterized by the consecutive emergence of different types of slow waves (Siclari et al., 2014; Spiess et al., 2018), and the EEG power maps and sources of several sub-bands in the delta range were differentially affected by SD during the first NREM sleep episode (Bersagliere et al., 2018). Such findings suggest the need for a more detailed characterization of the spatio-temporal dynamics of the different kinds of slow waves at SO after sustained wakefulness in future studies.

### Theta Activity

The spatio-temporal pattern of the theta power was mostly similar to that observed in the delta range, with a global power increase after SO and a progressive fronto-central consolidation, consistently with BSL findings (Marzano et al., 2013).

Electroencephalogram activity in the theta frequency range was one of the most affected by SD. Similar to the delta range, theta power exhibited a widespread increase of the post-SO changes in the REC compared to the BSL condition. Theta rhythm was prevalent in the oscillatory activity averaged across derivations after SO, differently from the BSL condition that was characterized by a maximum peak in the sigma range (Marzano et al., 2013). Moreover, we found a post-SO frontal enhancement in the theta oscillatory peak not observable at BSL (Marzano et al., 2013). An anterior involvement of the theta activity during the SO process in a REC night has been recently observed also with EEG source localization (Fernandez Guerrero and Achermann, 2019). The theta activity is considered a sensitive marker of homeostatic sleep pressure during wake (Finelli et al., 2000; Tinguely et al., 2006; De Gennaro et al., 2007; Vyazovskiy et al., 2011; Hung et al., 2013; Gorgoni et al., 2014), associated with impaired behavioral performance (Gorgoni et al., 2014; Bernardi et al., 2015; Fattinger et al., 2017; Nir et al., 2017) and characterized by a fronto-central maximum increase after SD interpreted as a higher recovery need in these areas (Horne, 1993; Finelli et al., 2001; Mander et al., 2010). The theta activity also exhibited a wide increase during REC sleep after sustained wakefulness, sharing with the delta activity a fronto-central predominance (Finelli et al., 2001; Marzano et al., 2010). During the SO process at BSL, the delta and theta activity showed similar “small world” features, interpreted as signs of functional disconnection (Vecchio et al., 2017). Together with these findings, our present results strongly support the notion of a crucial role of the theta activity during sleep (and not only during wake) as a biological marker of homeostatic sleep need.

Differently from the delta activity, however, an occipital maximum peak in both EEG power and oscillatory activity has been observed in our study, consistently with previous observations at SO in BSL (Wright et al., 1995; Marzano et al., 2013; Park et al., 2015). Moreover, occipital theta activity after SD showed a strong increase (secondary to the anterior one) during wake (De Gennaro et al., 2007; Gorgoni et al., 2014) and REC sleep (Finelli et al., 2001). Supported by findings in a single epileptic patient at SO (Marzano et al., 2013), we previously interpreted the scalp occipital theta oscillations after SO as a reflection of a peculiar local activity of the calcarine cortex, suggesting a possible involvement of this regional EEG feature in dreamlike mental activity and hypnagogic hallucinations, often observed during the falling-asleep process. Albeit no direct evidence for this hypothesis has been provided so far, recent findings with EEG source localization at SO seem to go in this direction (Fernandez Guerrero and Achermann, 2019).

### Alpha Activity

As observed in the BSL night (Hasan and Broughton, 1994; Tanaka et al., 1997; De Gennaro et al., 2001b; Marzano et al., 2013), also in the REC condition the alpha activity gradually declined before SO, and then increased during sleep, showing an inversion from a pre-SO posterior to a post-SO anterior prevalence. It has been proposed that such posterior-to-anterior shift may represent a modification of the functional meaning of the alpha activity during the falling-asleep process (Pivik and Harman, 1995; De Gennaro et al., 2004): the occipital prevalence in the pre-SO period should represent the typical “idle rhythm” of the relaxed eyes-closed wakefulness (Adrian and Matthews, 1934; Niedermeyer, 1997), while the post-SO fronto-central rise in alpha activity should be considered as part of the synchronization process, associated with sleep-maintaining mechanisms. Our findings indirectly corroborate this hypothesis: (a) the increase in the post- vs. pre-SO ratio in the REC condition compared to BSL in almost all the alpha frequency bins (with a specific fronto-central localization at 10 Hz) supports the existence of a relation between greater homeostatic sleep pressure and higher alpha activity during sleep, particularly in fronto-central areas; (b) the global post-SO increase in correspondence of the alpha oscillatory peak, not observed in normal condition (Marzano et al., 2013), suggests that the alpha activity at SO after prolonged wakefulness can be considered as a genuine rhythmic activity. Similarly, assessing EEG source localization, it has been observed a faster and higher increase of alpha activity after SO during a REC night, that progressively involve the frontal areas with increasing time (Fernandez Guerrero and Achermann, 2019). The authors proposed that this finding may be indicative of a genuine alpha increase instead of an enhancement of slow spindles (which mainly falls in the alpha frequency range) since spindle activity is usually reduced after sleep loss (De Gennaro and Ferrara, 2003). The greater SO-induced increase in the alpha frequency in REC compared to BSL observed in the present research supports this hypothesis.

### Sigma Activity

The sigma frequency range progressively increased after SO, exhibiting the centro-parietal predominance that characterizes sleep spindles (De Gennaro and Ferrara, 2003). This result partially represents a by-product of the methodological choice to set the beginning of sleep in correspondence of the first epoch of stage 2 (i.e., the emergence of sleep spindles and/or K-complexes). However, similar findings have been observed in a BSL night by using a different method to define the timing of the SO period (Siclari et al., 2014).

Notably, no differences between BSL and REC have been found in the sigma power post- vs. pre-SO ratio. A reduction of spindle/sigma activity has been previously observed in a REC night after different protocols of SD (Borbély et al., 1981; De Gennaro et al., 2000; Curcio et al., 2003; De Gennaro and Ferrara, 2003; Marzano et al., 2010). Moreover, tracking the temporal dynamics of brain activity at SO with EEG source localization, Fernandez Guerrero and Achermann (2019) recently found a reduced ability to generate sigma activity after SD. However, they considered a different timing to define SO (2 min pre- vs. 10 min post-SO). It is plausible that the post-SD spindle reduction may not be observed comparing the shorter post-sleep intervals considered in our study (5 min pre- and post-SO).

The detection of the oscillatory activity showed a global increase of the sigma rhythm after SO, mostly confirming BSL findings (Marzano et al., 2013) albeit with a lower oscillatory peak (BSL: 13.00 Hz; REC: 12.12 Hz) that seems consistent with the previously observed reduction of the spindle frequency during REC sleep (Knoblauch et al., 2003; Olbrich et al., 2014).

### Beta Activity

The frequency bins ≥ 18 Hz were characterized by a progressive and generalized decrease during the falling-asleep process, that begins before the SO without a specific topographical predominance, extending previous observation at BSL and representing an arousal reduction (Merica et al., 1991; Merica and Gaillard, 1992; De Gennaro et al., 2001a; Marzano et al., 2013).

No significant changes in the REC condition compared to BSL were observed for the post- vs. pre-SO ratio. The influence of SD on the EEG power during the falling-asleep process, then, seems to be expressed more in term of higher deactivation (i.e., stronger synchronization) than reduced activation. Consistently, analyzing the cortical sources of the beta activity at SO, Fernandez Guerrero and Achermann (2019) found that less that 10% of the voxels differed between BSL and REC conditions, although the reduction of the beta activity was faster in REC.

Albeit the spatio-temporal modulation of the oscillatory activity in the beta frequency pointed to a progressive reduction in this frequency range, the comparison between pre- and post-SO periods in the beta frequency peak showed an increase in the midline centro-parietal derivation, not observed during the BSL night (Marzano et al., 2013). This result is surprising (a) considering the parallel decrease in the EEG beta power, (b) starting from the evidence of a beta activity reduction at SO (Merica et al., 1991; De Gennaro et al., 2001a; Marzano et al., 2013) (c) bearing in mind that beta activity is usually considered an electrophysiological marker of arousal and motor/cognitive functioning (Basar-Eroglu et al., 1996; Neuper and Pfurtscheller, 2001; Kilavik et al., 2013; Merker, 2013).

On the other hand, an increase in beta power during REC sleep has been observed after SWS deprivation (Ferrara et al., 2002) and (albeit not significantly) after total SD (Marzano et al., 2010). It should be considered that fast frequencies are also generated during the depolarizing phase of the slow sleep oscillations, and not only during activation processes (Steriade, 2001). Moreover, analyzing the EEG dynamics during active and quite wake in rats, Grønli et al. (2016) found that during active wakefulness the beta (and gamma) activity was increased, while in quite wakefulness the beta activity paralleled the delta and theta activity in tracking the sleep need, with consistent modifications of the cortical lactate concentration (a measure of cerebral glucose utilization). Albeit these findings do not give an elucidation about the increase of the beta rhythm observed in our study, they point to the need for further characterization of state-dependent beta oscillations.

## Conclusion

The interest for the characterization of the local brain activity during the SO process is growing, and our findings appear complementary to recent observations on the effect of SD on the wake-sleep transition assessed with EEG source localization and effective connectivity (Fernandez Guerrero and Achermann, 2018, 2019). Beyond confirming the local nature of the falling-asleep process, our results provide for the first time a window on the spatio-temporal dynamics of the EEG power spectra and oscillatory activity at SO after a period of prolonged wakefulness. We found that SD (a) affected the EEG power at SO, increasing the magnitude of SO-related changes in the frequencies ≤10 Hz, (b) induced a predominant theta oscillatory rhythm, and (c) promoted an earlier appearance of sleep-related rhythmic oscillations like alpha and frontal theta, not observed at BSL, enhancing the concordance between FFT and BOSC.

From a methodological standpoint, our results confirm the BOSC method as a useful tool to characterize the EEG spatiotemporal dynamics during different states of consciousness (and the transition between them), providing information on the genuine rhythmic nature of the electrophysiological activities observed.

Finally, we must remember that SD is extremely diffuse, and its price in terms of working and car accidents is dramatically high. The characterization of the electrophysiological processes during the SO period after sustained wakefulness could represent an essential contribute to help the prevention of the damages provoked by sleep loss, e.g., detecting the possible frequency-specific spatio-temporal targets for brain stimulation protocols aimed at the promotion of vigilance and the reduction of sleepiness (Annarumma et al., 2018).

## Data Availability

The datasets generated for this study are available on request to the corresponding author.

## Author Contributions

MG, LDG, and MF: substantial contributions to the conception and design of the work, interpretation of data, and drafting the work and revising it critically for important intellectual content. MG, CB, ADA, SS, CM, and FM: acquisition and analysis of data. MG, CB, ADA, SS, CM, FM, MF, and LDG: final approval of the paper and agreement to be accountable for all aspects of the work in ensuring that questions related to the accuracy or integrity of any part of the work are appropriately investigated and resolved.

## Conflict of Interest Statement

The authors declare that the research was conducted in the absence of any commercial or financial relationships that could be construed as a potential conflict of interest.
